# Supplementary material for: A Ropy Exopolysaccharide-Producing Strain Bifidobacterium pseudocatenulatum Bi-OTA128 Alleviates Dextran Sulfate Sodium-Induced Colitis in Mice
Source: Nutrients. 2023 Dec 1;15(23):4993. doi: 10.3390/nu15234993 (PMC10707796; doi:10.3390/nu15234993)
Supplement: Supplementary file 1 [file nutrients-15-04993-s001.zip › nutrients-2695550-supplementary.pdf]

A Ropy Exopolysaccharide-Producing Strain *Bifidobacterium pseudocatenulatum* Bi-OTA128 Alleviates Dextran Sulfate Sodium-Induced Colitis in Mice

Hui Wang [1], Xinyuan Zhang [1], Xinfang Kou [1], Zhengyuan Zhai [1,*] and Yanling Hao [2,3,*]

[1] College of Food Science and Nutritional Engineering, China Agricultural University, Beijing 100083, China
[2] Key Laboratory of Precision Nutrition and Food Quality, Department of Nutrition and Health, China Agricultural University, Beijing 100083, China
[3] Food Laboratory of Zhongyuan, Luohe 462300, China
[*] Correspondence: zhaizy@cau.edu.cn (Z.Z.); haoyl@cau.edu.cn (Y.H.)

Materials and Methods

Isolation and purification of EPS from *B. pseudocatenulatum* Bi-OTA128

The isolation of EPS from *B. pseudocatenulatum* Bi-OTA128 was performed as follows. After incubation at 37°C for 3 days, the biomass of Bi-OTA128 was collected from the surface of agar-MRSc plates by adding ultrapure water. The ultrasonic treatment at 50W power in 3-5 s pulses for 6 min was performed to release EPS from bacterial surface. Then cell-free supernatants were collected after centrifugation at 12,000×g, 4°C for 30 min. The proteins in supernatants were precipitated by adding trichloroacetic acid (TCA) at final concentration of 4% (v/v). After 2 h incubation at 4°C, the supernatants were collected by centrifugation at 6,000×g for 15 min. Subsequently, three volumes of cold absolute ethanol were added and kept at 4°C for 2 days to precipitate crude EPS. Finally, the EPS pellet was collected by centrifugation at 3,000×g, 4°C for 10 min, dialyzed against distilled water (Mw cut-off: 8,000-14,000 Da) at 4°C for 3 days, and lyophilized to obtain a crude EPS.

DNase (2.5 μg/mL) and Pronase E (50 μg/mL) were applied to remove nucleic acid and protein in crude EPS by sequentially incubated at 37°C for 6 h. The enzymes were further removed by TCA precipitation. EPS solution (2 mg/mL in ultrapure water) was then subjected to a DEAE-Sepharose Fast Flow anion exchange column (1.0×30 cm, Solarbio) and eluted with a step gradient NaCl solution (0, 0.1, 0.3, and 0.5 M) at flow rate of 1 mL/min. The eluted fractions belonging to single absorption peaks were collected according to the elution curve plotting by phenol-sulfuric acid assay (detected at 490 nm). The purified EPS component was then dialyzed and lyophilized.

Assessment of EPS Produced by *B. pseudocatenulatum* Bi-OTA128 on LPS-Induced Inflammation in RAW 264.7 Cells

The murine macrophage cell line RAW 264.7 was obtained from MeisenCTCC (Zhejiang, China) and cultured in Dulbecco's modified Egle medium (DMEM; Gibco, USA) which was supplemented with 10% (v/v) fetal bovine serum and 1% penicillin/streptomycin at 37°C in a 5% CO_2 humidified incubator. Cells were sub-cultured over three passages and harvested when it reached 70-80% confluency.

RAW264.7 macrophages (5×10^4 cells per well) were pre-incubated in 96-well plates for 14 h. After washed twice with phosphate buffered saline (PBS, pH 7.2), cells were simultaneously treated with 1 μg/mL lipopolysaccharide (LPS) and EPS at concentrations of 50, 100, 200, and 400 μg/mL (dissolved in DMEM medium) and incubated at 37°C, 5% CO_2 for 24 h. The DMEM medium and only LPS-treated cells were used as blank and positive control, respectively. After incubation, the levels of nitric oxide (NO) production and cytokines TNF-α, IL-1β, and IL-6 in supernatants were determined using NO detection kit (Elabscience Biotechnology Co. Ltd, Wuhan, China) or commercially procured ELISA kits (Elabscience) in accordance with manufacturer's instructions.

Table S1. Disease activity index (DAI) scoring system

Score	Body weight change [a]	Stool Consistency	Stool Occult Blood [b]
0	None body weight loss	Granular hard stools	No stool occult blood
1	1–5% body weight loss	Soft stools, granular shapes	Color change from light green to green within 10 s
2	6–10% body weight loss	Very soft, shapeless stools	Color change from green to blue within 30 s
3	11–18% body weight loss	Watery stools (Diarrhea)	Color change from green to dark blue immediately
4	> 18% body weight loss		Visible rectal bleeding

[a] Body weight change was compared with the body weight at Day 14 before DSS treatment.

[b] The hemoccult positive degree was assessed by using the Fecal Occult Blood Test Kit (Brybio, Beijing, China) based on color change.

Table S2. Histological scores of colon damage

Score	Inflammation severity	Inflammation extent	Crypt damage
0	None inflammation	None inflammation	Intact crypt, no damage
1	Mild inflammation with slight lesion sites	Inflammation observed only in mucosa	Basal 1/3 crypt damaged
2	Moderate inflammation with several lesion sites	Inflammation observed in mucosa and submucosa positions	Basal 2/3 crypt damaged
3	Severe inflammation with whole colonic damage	Inflammation observed in transmural colonic tissues	Crypt lost but surface epithelium present
4			Crypt and surface epithelium lost

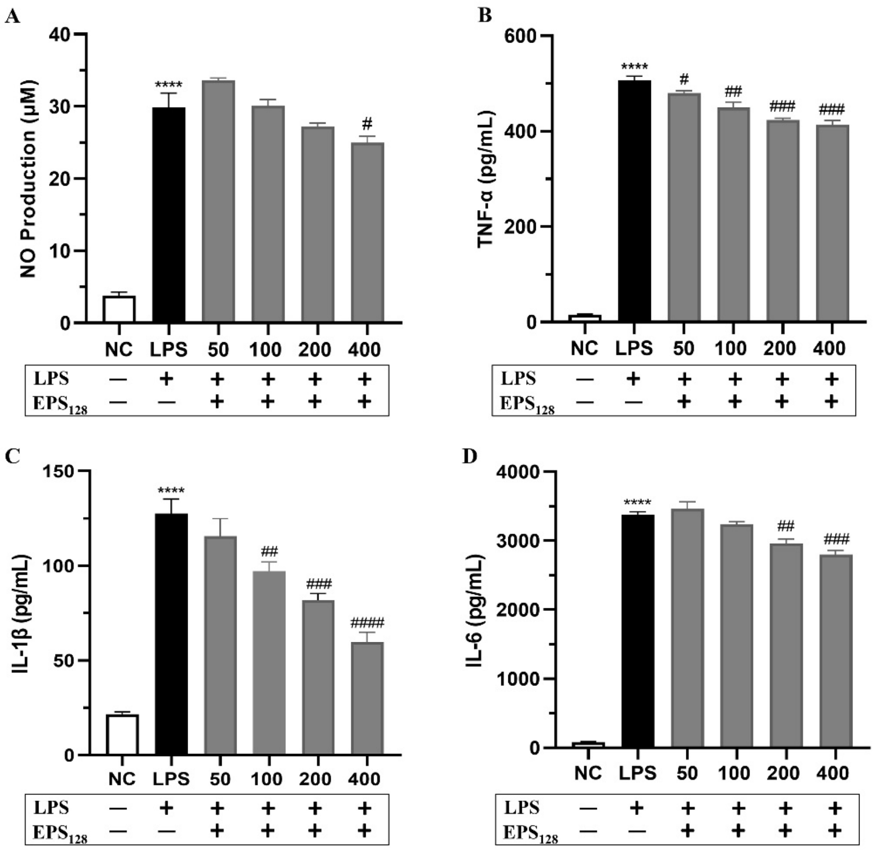

Figure S1. Effects of EPS produced by *B. pseudocatenulatum* strain Bi-OTA128 on inflammatory cytokines production in LPS-induced RAW264.7 cells. (**A**) NO production, (**B**) TNF-α, (**C**) IL-1β, and (**D**) IL-6 levels in culture supernatants after incubation at 37°C, 5% CO_2 for 24 h. n = 3. **** $p < 0.0001$ compared with the NC group. # $p < 0.05$, ## $p < 0.01$, ### $p < 0.001$, and #### $p < 0.0001$ showed significant difference compared with the LPS group. NC, negative control group; LPS, lipopolysaccharide.

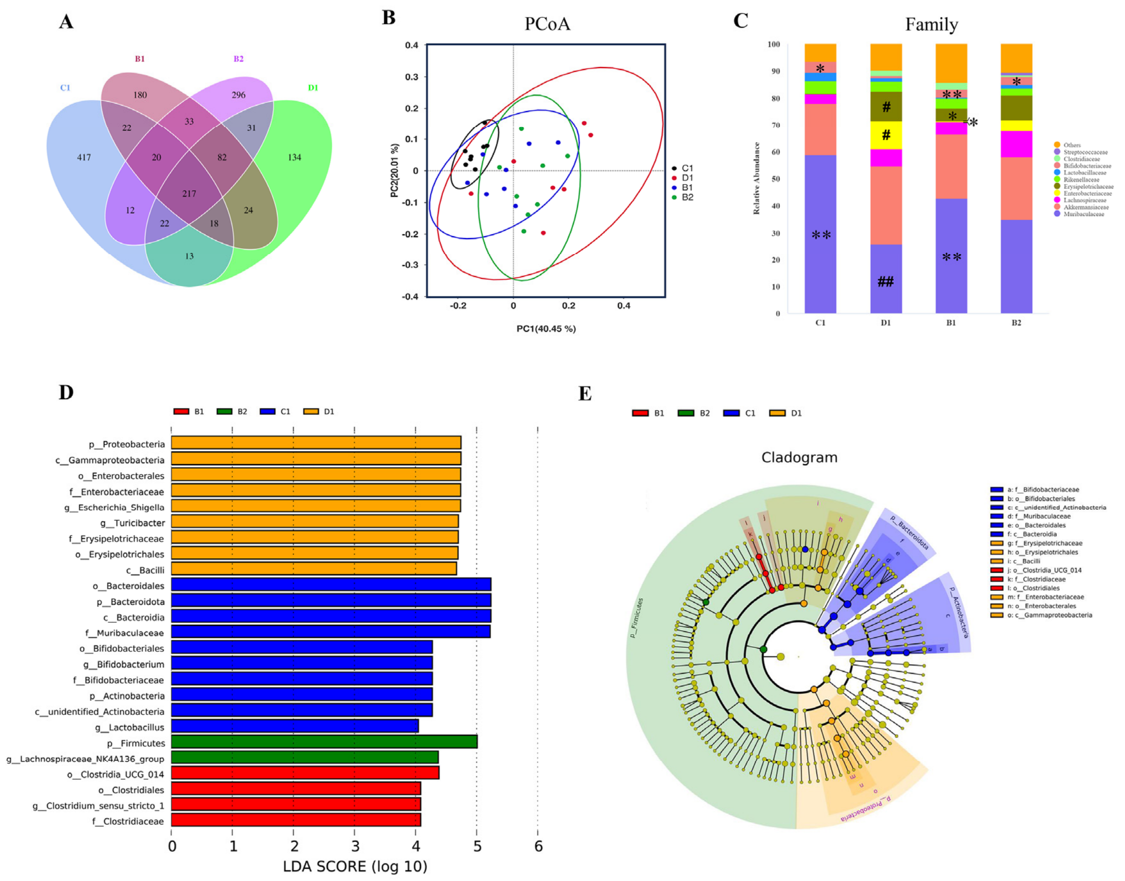

Figure S2. Effects of *B. pseudocatenulatum* intervention on gut microbiota composition in DSS-induced colitis in mice. (**A**) Veen diagram; (**B**) Principal coordinate analysis (PCoA); (**C**) Relative abundance of microbiota in the Family level. # $p < 0.05$ and ## $p < 0.01$ represent comparison with the C1 (Control) group; * $p < 0.05$ and ** $p < 0.01$ represent the comparison with the D1 (DSS) group. (**D**,**E**) LDA Effect Size (LEfSe) analysis for differentially abundant taxa. The LDA score > 4.0. Statistics was based on amplicon sequence variants (ASVs) and analyzed by QIIME2 platform.
